# Colonization factor CS30 from enterotoxigenic *Escherichia coli* binds to sulfatide in human and porcine small intestine

**DOI:** 10.1080/21505594.2020.1749497

**Published:** 2020-04-14

**Authors:** Astrid Von Mentzer, Dani Zalem, Zofia Chrienova, Susann Teneberg

**Affiliations:** a Department of Microbiology and Immunology, Sahlgrenska Academy, Institute of Biomedicine, University of Gothenburg, Gothenburg, Sweden; b Wellcome Sanger Institute: Parasites and Microbes Programme, Hinxton, UK; c Department of Medical Biochemistry and Cell Biology, Sahlgrenska Academy, Institute of Biomedicine, University of Gothenburg, Gothenburg, Sweden; d Department of Chemistry, Faculty of Science, University of Hradec Králové, Hradec Králové, Czech Republic

**Keywords:** Enterotoxigenic *E. coli*, colonization factor CS30, microbial adhesion, carbohydrate binding, glycosphingolipid characterization, sulfatide

## Abstract

The ability to adhere via colonization factors to specific receptors located on the intestinal mucosa is a key virulence factor in enterotoxigenic *Escherichia coli* (ETEC) pathogenesis. Here, the potential glycosphingolipid receptors of the novel human ETEC colonization factor CS30 were examined by binding of CS30-expressing bacteria to glycosphingolipids on thin-layer chromatograms. We thereby found a highly specific binding of CS30-expressing bacteria to a fast-migrating acid glycosphingolipid of human and porcine small intestine, while no binding was obtained with a mutant ETEC strain unable to express CS30 fimbriae. The CS30 binding glycosphingolipid from human small intestine was isolated and characterized by mass spectrometry as sulfatide (SO_3_-3Galβ1Cer). Comparative binding studies using sulfatides with different ceramide compositions gave a preferential binding of CS30 to sulfatide with d18:1-h24:0 ceramide. This ceramide species of sulfatide was also isolated from human small intestine and characterized by mass spectrometry and antibody binding. These studies implicate sulfatide as candidate receptor for mediating attachment of CS30-fimbriated ETEC to human and porcine small intestinal cells. Our findings may be a basis for designing receptor saccharide analogues for inhibition of the intestinal adhesion of CS30-expressing *E. coli*.

## Introduction

Diarrheal disease is a leading cause of child mortality and morbidity in the world according to the World Health Organization (WHO). The enteric pathogen enterotoxigenic *Escherichia coli* (ETEC) is the most common cause of bacterial diarrhea in children, mainly in resource-poor regions where access to clean water and proper sanitation are limited [1], and in travelers to endemic areas [2]. Diarrhea due to ETEC infection is considered the most common cause in offspring of some farm animals, such as piglets and calves [3,4]. Improved surveillance systems and robust diagnostics tools are needed to be able to properly estimate the true burden of ETEC disease in both humans and livestock [1,5].

Living in close proximity with domestic livestock and poultry is more common in resource-poor countries where animal husbandry serves as a primary source of income. Livestock and domestic animals are common sources of fecal contamination of water and in households [6]. Thus, living with livestock increases the risk of fecal contamination and subsequently elevates the risk of diarrheal pathogen transmission between animals and humans. Furthermore, it has been shown that livestock exposure is associated with diarrheal illness in humans, mainly through fecal contamination of the household environment [7].

ETEC is characterized by the ability to produce enterotoxins and outer membrane proteins, called colonization factors (CFs) for adherence to the intestinal cells that enables colonization of the small intestine. The CFs recognize specific receptors and are considered host-specific. Interestingly, a relatively new class of CFs identified in human-associated ETEC, Class 1B, encompassing CS12, CS18, CS20, and CS30 are related to the adhesin F6 (987P), which is expressed by ETEC infecting neonatal piglets [8,9].

Many of these CFs have tip-localized adhesins which recognize carbohydrate receptors to mediate colonization of host target tissue. Several such glycosphingolipid receptors have been characterized for adhesins from ETECs infecting both humans [10,11] and pigs [12–15].

The recently identified CF CS30 was found in ETEC isolates collected from children with diarrhea worldwide. The operon structure of CFs belonging to Class 1b is highly conserved and the same structure is seen in the operon of the porcine CF F6 (987P) [9]. The major subunit of CS30 (CsmA) has more than 50% amino acid homology with the major subunit of F6 (FasA) [9].

In the present study, the potential carbohydrate recognition by CS30 was investigated by binding of CS30 expressing ETEC to glycosphingolipids from various sources on thin-layer chromatograms. A distinct binding to a fast-migrating acid glycosphingolipid of human and porcine small intestine was found. The CS30 binding glycosphingolipid from human small intestine was isolated and characterized by mass spectrometry as sulfatide (SO_3_-3Galβ1Cer). Binding studies using sulfatides with different ceramide species demonstrated a preferential binding to sulfatide with d18:1-h24:0 ceramide, which was one of the ceramide species of sulfatide isolated from human small intestine.

## Materials and methods

### Bacterial strains, culture conditions, and labeling

The wild type CS30 expressing ETEC strain E873 was cultured on CFA agar plates containing 0.15% crude bile over night at 37°C. Thereafter, bacteria were added to CFA broth containing 0.15% crude bile and cultured for 3 h at 37°C. For metabolic labeling, the medium (10 ml) was supplemented with 10 μl ^35^S-methionine (400 μCi; PerkinElmer; NEG77207MC). The bacteria were harvested by centrifugation, washed three times with PBS (phosphate-buffered saline, pH 7.3), and resuspended in PBS containing 2% (w/v) bovine serum albumin, 0.1% (w/v) NaN_3_, and 0.1% (w/v) Tween 20 (BSA/PBS/TWEEN) to a bacterial density of 1 × 10^8^ CFU/ml. Attempts to purify CS30 using methods that were previously used for purification of other CFs [16–19] were not successful. Therefore, the binding studies were done using the CS30 wild type strain.

The same conditions, with addition of kanamycin 0.05 mg/ml, were used for culture and labeling of the mutant ETEC strain E873Δ*csmA* with disrupted *csmA* (major subunit) gene.

### Reference glycosphingolipids

Total acid and nonacid glycosphingolipid fractions were isolated as described [20]. Individual glycosphingolipids were isolated by repeated chromatography on silicic acid columns and by HPLC, and identified by mass spectrometry [21,22] and ^1^H-NMR spectroscopy [23].

### Thin-layer chromatography

Thin-layer chromatography was done on glass- or aluminum-backed silica gel 60 high performance thin-layer chromatography plates (Merck; 105641/105547). Glycosphingolipid mixtures (40 μg), or pure glycosphingolipids (2–4 μg), were applied to the plates, and eluted with chloroform/methanol/water 60:35:8 (by volume), or chloroform/methanol/water 65:25:4 (by volume). Chemical detection was done with anisaldehyde [24].

### Chromatogram binding assays

Binding of radiolabeled bacteria to glycosphingolipids on thin-layer chromatograms was done as described [10]. Dried chromatograms were dipped in diethylether/*n*-hexane (1:5 v/v) containing 0.5% (w/v) polyisobutylmethacrylate for 1 min. The chromatograms were blocked with BSA/PBS/TWEEN for 2 h at room temperature. Thereafter the plates were incubated for 2 h at room temperature with ^35^S-labeled bacteria (1–5 × 10^6^ cpm/ml) diluted in BSA/PBS/TWEEN. After washing six times with PBS, and drying, the plates were autoradiographed for 12–36 h using x-ray films (Carestream; 8941114).

Chromatogram binding assays with monoclonal antibodies directed against SO_3_-3Galβ (Sulf-1 antibody) were done as described [25].

### Isolation of the CS30 binding glycosphingolipid from human small intestine

A total acid glycosphingolipid fraction (43.2 mg) from human small intestine was first separated on a 10 g Iatrobeads column (Iatron Laboratories Inc.; 6RS-8060) eluted with chloroform/methanol/water (60:35:8, by volume), 30 × 1 ml. The fractions obtained were analyzed by thin-layer chromatography and anisaldehyde staining, and pooled into four subfractions according to mobility on the thin-layer chromatograms. The *E. coli* CS30 binding activity of these four fractions was assessed using the chromatogram binding assay. After this first separation, one fraction (18.6 mg) containing the CS30 binding fast-migrating compounds was obtained. This fraction was further separated on a second 10 g Iatrobeads column eluted with chloroform/methanol/water (60:35:8, by volume), 40 × 0.5 ml. Again the fractions obtained were analyzed by thin-layer chromatography and anisaldehyde staining, and pooled into three subfractions according to mobility on the thin-layer chromatograms. The first and the third fractions obtained had distinctly different mobility on thin-layer chromatograms indicating different ceramide compositions. In order to isolate pure ceramide species to test for CS30 binding, these two fractions (approximately 4 mg each) were further separated on 10 g Iatrobeads columns, eluted, and pooled as above.

### Isolation of sulfatides with different ceramide composition

The strategy described above was also used for separation of sulfatides with different ceramides from human stomach, and from human meconium.

### LC-ESI/MS of glycosphingolipids

The isolated glycosphingolipid containing fractions were analyzed by LC-ESI/MS as described [26]. Aliquots of the glycosphingolipid fractions were dissolved in methanol:acetonitrile in proportion 75:25 (by volume) and separated on a 100 × 0.250 mm column, packed in-house with 5 μm polyamine II particles (YMC Europe GmbH; PB12S05). An autosampler, HTC-PAL (CTC Analytics AG), equipped with a cheminert valve (0.25 mm bore) and a 2 µl loop, was used for sample injection. An Agilent 1100 binary pump (Agilent Technologies) delivered a flow of 250 µl/min, which was split down in an 1/16” microvolume-T (0.15 mm bore) (Vici AG International) by a 50 cm × 50 µm i.d. fused silica capillary before the injector of the autosampler, allowing approximately 2–3 µl/min through the column. Samples were eluted with an aqueous gradient (A: 100% acetonitrile to B: 10 mM ammonium bicarbonate). The gradient (0–50% B) was eluted for 40 min, followed by a wash step with 100% B, and equilibration of the column for 20 min. The samples were analyzed in negative ion mode on a LTQ linear quadropole ion trap mass spectrometer (Thermo Electron), with an IonMax standard ESI source equipped with a stainless steel needle kept at –3.5 kV. Compressed air was used as nebulizer gas. The heated capillary was kept at 270°C, and the capillary voltage was –50 kV. Full scan (*m*/*z* 500–1800, two microscan, maximum 100 ms, target value of 30,000) was performed, followed by data-dependent MS^2^ scans (two microscans, maximun 100 ms, target value of 10.000) with normalized collision energy of 35%, isolation window of 2.5 units, activation *q* = 0.25 and activation time 30 ms). The threshold for MS^2^ was set to 500 counts.

Data acquisition and processing were conducted with Xcalibur software (Version 2.0.7). Manual assignment of glycosphingolipid sequences was done with the assistance of the Glycoworkbench tool (Version 2.1), and by comparison of retention times and MS^2^ spectra of reference glycosphingolipids.

### Inhibition studies

To test for inhibition of binding, CS30 expressing *E. coli* were incubated with various concentrations (10 mg/ml, 5 mg/ml, 1 mg/ml, and 0.1 mg/ml) of saccharides and anionic polysaccharides in PBS. Incubations were done for 2 h at room temperature, and thereafter the suspensions were diluted 40 times, and used in the chromatogram binding assays, as described above. Dextran sulfate (265152–10), and dextran (381092P) were from VWR International, while sodium octadecyl-sulfate (293946–1 G), and galactose 4-sulfate (90572), were from Sigma-Aldrich.

## Results

### Screening for CS30 carbohydrate recognition

**Figure 1.**
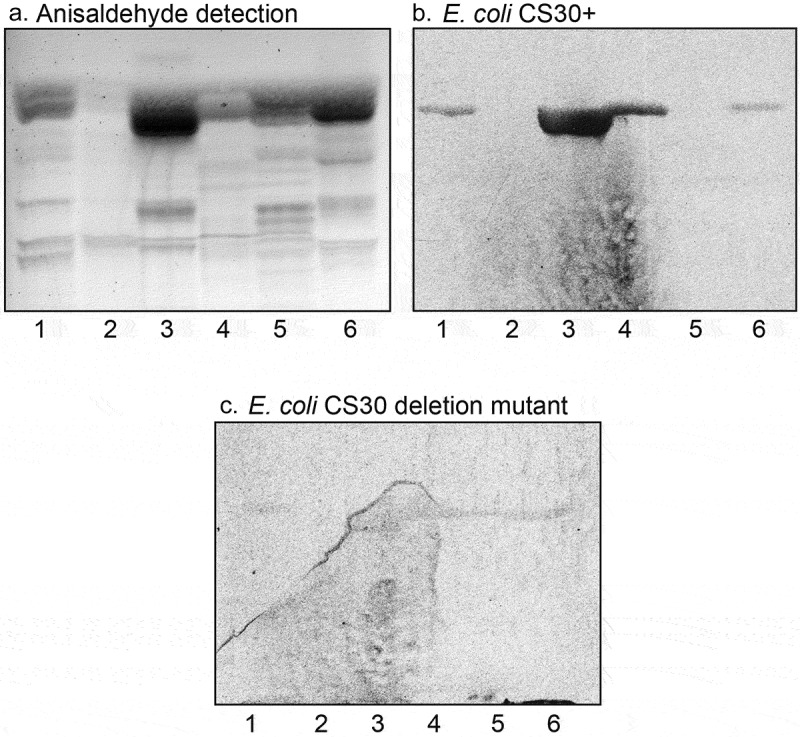
Screening for CS30 carbohydrate recognition. Thin-layer chromatogram detected with anisaldehyde (a), and autoradiograms obtained by binding of CS30 expressing *E. coli* strain E873 (b), and the mutant *E. coli* strain E873Δ*csmA*, lacking the major subunit CsmA (c). The glycosphingolipids were separated on aluminum-backed silica gel plates, using chloroform/methanol/water 60:35:8 (by volume) as solvent system, and the binding assays were performed as described under “Materials and methods.” Autoradiography was for 12 h. The lanes were: Lane 1, total acid glycosphingolipids of human small intestine, 40 μg; Lane 2, gangliosides of human small intestine, 40 μg; Lane 3, total acid glycosphingolipids of human meconium, 40 μg; Lane 4, total acid glycosphingolipids of rabbit small intestine, 40 μg; Lane 5, total acid glycosphingolipids of monkey small intestine, 40 μg; Lane 6, total acid glycosphingolipids of cat small intestine, 40 μg.

**Figure 2.**
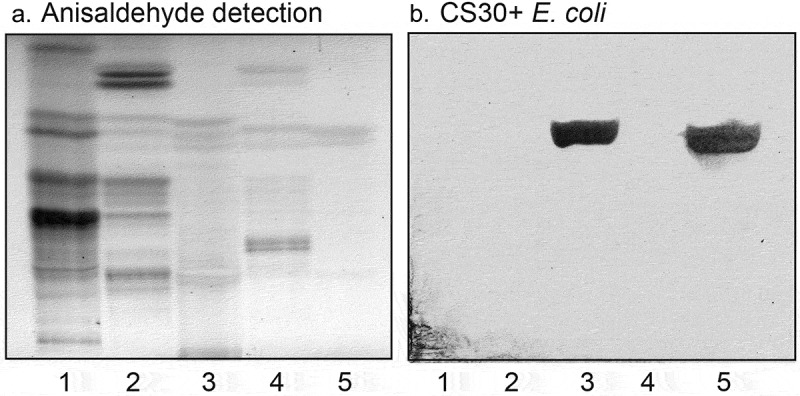
Binding of CS30 expressing *E. coli* to glycosphingolipids of human and porcine small intestine. Thin-layer chromatogram detected with anisaldehyde (a), and autoradiogram obtained by binding of the CS30 expressing *E. coli* strain E873 (b). The glycosphingolipids were separated on aluminum-backed silica gel plates, using chloroform/methanol/water 60:35:8 (by volume) as solvent system, and the binding assays were performed as described under “Materials and methods.” Autoradiography was for 12 h. The lanes were: Lane 1, reference nonacid glycosphingolipids of human erythrocytes blood group AB, 40 μg; Lane 2, nonacid glycosphingolipids of human small intestine, 40 μg; Lane 3, acid glycosphingolipids of human small intestine, 40 μg; Lane 4, nonacid glycosphingolipids of porcine small intestine, 40 μg; Lane 5, acid glycosphingolipids of porcine small intestine, 40 μg.

In order to test for CS30 carbohydrate recognition, we examined the binding of wild type CS30-expressing ETEC strain E873, and the mutant ETEC strain E873Δ*csmA* with disruption of the major subunit, to a number of total acid and total nonacid glycosphingolipid fractions from various sources available in our glycosphingolipid bank (exemplified in Figure 1(a)). Thereby, the bacteria were exposed to a large number of variant carbohydrate structures. During these studies we repeatedly observed a selective binding of the CS30 expressing ETEC to a fast-migrating compound in acid glycosphingolipid fractions (exemplified in Figure 1(b), lanes 1, 3, 4, and 6; Figure 2(b), lanes 3 and 5). Binding to the fast-migrating compound in acid glycosphingolipid fractions was not obtained with the mutant ETEC strain unable to express CS30 (Figure 1(c)). There was no binding of the CS30 positive bacteria to the more slow-migrating compounds (mainly gangliosides) in the acid fractions, or to any nonacid glycosphingolipids (exemplified in Figure 2(a,b), lanes 1, 2 and 4).

**Figure 3.**
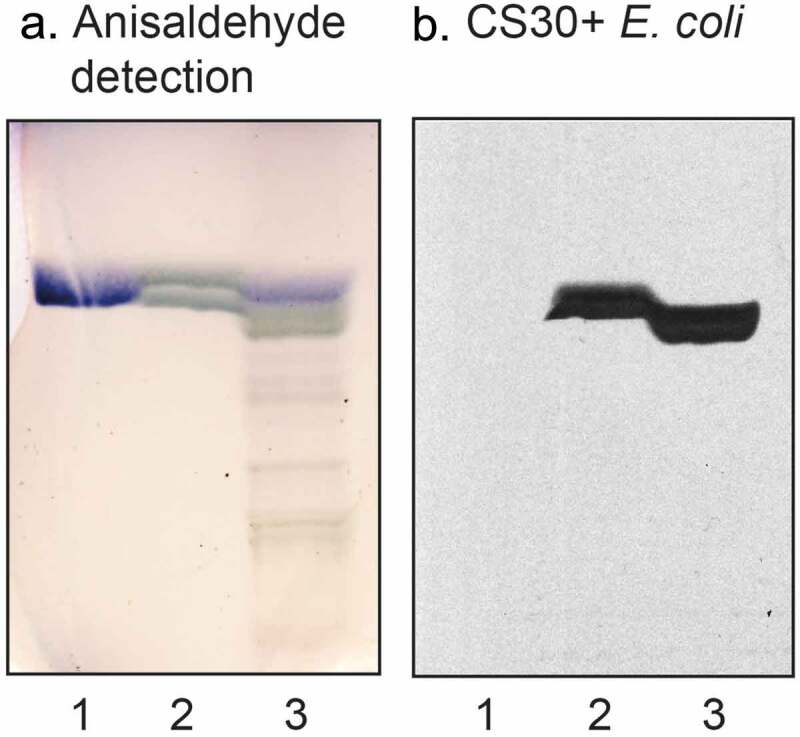
Binding of CS30 expressing *E. coli* to glycosphingolipids of human small intestine. Thin-layer chromatogram detected with anisaldehyde (a), and autoradiogram obtained by binding of the CS30 expressing *E. coli* strain E873 (b). The glycosphingolipids were separated on aluminum-backed silica gel plates, using chloroform/methanol/water 60:35:8 (by volume) as solvent system, and the binding assays were performed as described under “Materials and methods.” Autoradiography was for 12 h. The lanes were: Lane 1, reference cholesterol sulfate, 4 μg; Lane 2, sulfatide (SO_3_-Galβ1Cer), 4 μg; Lane 3, total acid glycosphingolipids of human small intestine, 40 μg.

Thus, the CS30 expressing ETEC bound to a fast-migrating compound in acid glycosphingolipid fractions of human and porcine small intestine (Figure 1(b), lane 1; Figure 2(b), lanes 3 and 5). In this region of human small intestine, there are two co-migrating compounds, cholesterol sulfate and sulfatide (SO_3_-3Galβ1Cer) [27]. When binding to reference compounds was tested the CS30 positive bacteria bound to sulfatide (Figure 3(a,b), lane 2), whereas cholesterol sulfate was not recognized (Figure 3(a,b), lane 1).

### Isolation and characterization of the CS30 binding glycosphingolipid from human small intestine

Next the fast-migrating glycosphingolipid recognized by CS30 expressing ETEC was isolated from the total acid fraction of human small intestine by a series of Iatrobeads column chromatographies. Fractions obtained were pooled according to mobility on thin-layer chromatograms, and binding of CS30 positive ETEC. Finally, three CS30 binding subfractions were obtained, denoted fractions HI-1, HI-2, and HI-3.

**Figure 4.**
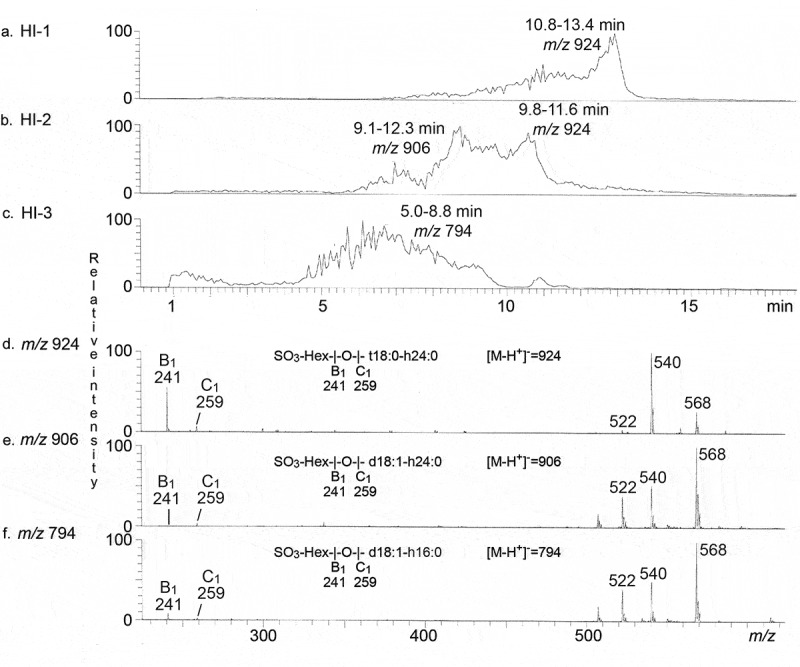
LC-ESI/MS characterization of CS30 binding glycosphingolipids isolated from human small intestine. (a) Base peak chromatogram from LC-ESI/MS of fraction HI-1. (b) Base peak chromatogram from LC-ESI/MS of fraction HI-2. (c) Base peak chromatogram from LC-ESI/MS of fraction HI-3. (d) MS^2^ of the ion at *m*/*z* 924 in (A) (retention time 12.6 min). (e) MS^2^ of the ion at *m*/*z* 906 in (B) (retention time 8.8 min). (f) MS^2^ of the ion at *m*/*z* 794 in (C) (retention time 6.5 min).

The isolated CS30 binding fractions were first characterized by LC-ESI/MS (exemplified in Figure 4). The three base peak chromatograms (Figure 4(a–c)) had molecular ions at *m*/*z* 924, *m*/*z* 906 and *m*/*z* 794, corresponding to glycosphingolipids with a sulfated hexose (SO_3_-Hex) and t18:0-h24:0, d18:1-h24:0 and d18:1-h16:0 ceramides, respectively. The three MS^2^ spectra (Figure 4(d–f)) all had a B_1_ ion at *m*/*z* 241, and a C_1_ ion at *m*/*z* 259, demonstrating a terminal SO_3_-Hex. The ions at *m*/*z* 522, 540 and 568 are due to loss of the fatty acyl from the molecular ion [28].

**Figure 5.**
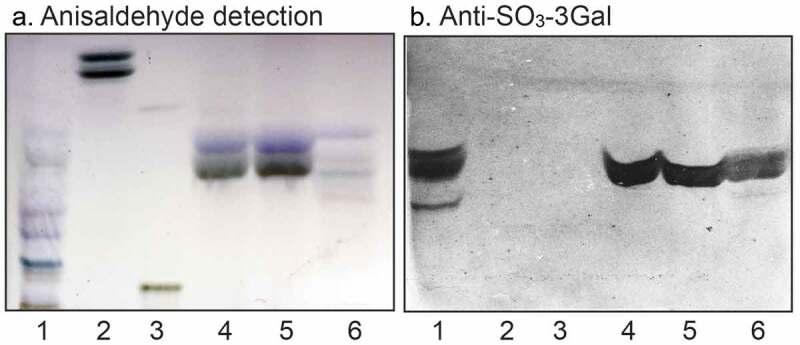
Binding of monoclonal antibodies directed toward SO_3_-3Galβ to the acid glycosphingolipid subfractions from human small intestine. Thin-layer chromatogram after detection with anisaldehyde (a), and autoradiogram obtained by binding of anti-SO_3_-3Galβ antibodies (b). The glycosphingolipids were separated on aluminum-backed silica gel plates, using chloroform/methanol/water 60:35:8 (by volume) as solvent system, and the binding assays were performed as described under “Materials and methods.” Autoradiography was for 12 h. The lanes were: Lane 1, reference acid glycosphingolipids of moose large intestine, 40 μg; Lane 2, reference galactosylceramide (Galβ1Cer), 4 μg; Lane 3, reference sulf-gangliotetraosylceramide (SO_3_-3Galβ3GalNAcβ4Galβ4Glcβ1Cer), 4 μg; Lane 4, sulfatide with d18:1-h24:0 and t18:0-h24:0 ceramides (fraction HI-2), 4 μg; Lane 5, sulfatide with t18:0-h24:0 ceramide (fraction HI-1), 4 μg; Lane 6, sulfatide with d18:1-h16:0 ceramide (fraction HI-3), 4 μg.

Binding of monoclonal antibodies directed toward SO_3_-3Galβ to the three fractions isolated from human small intestine was also tested. The antibodies bound to all three fractions (Figure 5b), confirming the sulfatide content deduced from mass spectrometry.

Thus, the acid glycosphingolipid fractions of human small intestine recognized by CS30 positive *E. coli* all contained sulfatide. Fraction HI-1 had predominantely t18:0-h24:0 ceramide, fraction HI-2 a mixture of t18:0-h24:0 and d18:1-h24:0, and fraction HI-3 had mainly d18:1-h16:0 ceramide.

### Isolation and characterization of sulfatides with variant ceramide composition

**Figure 6.**
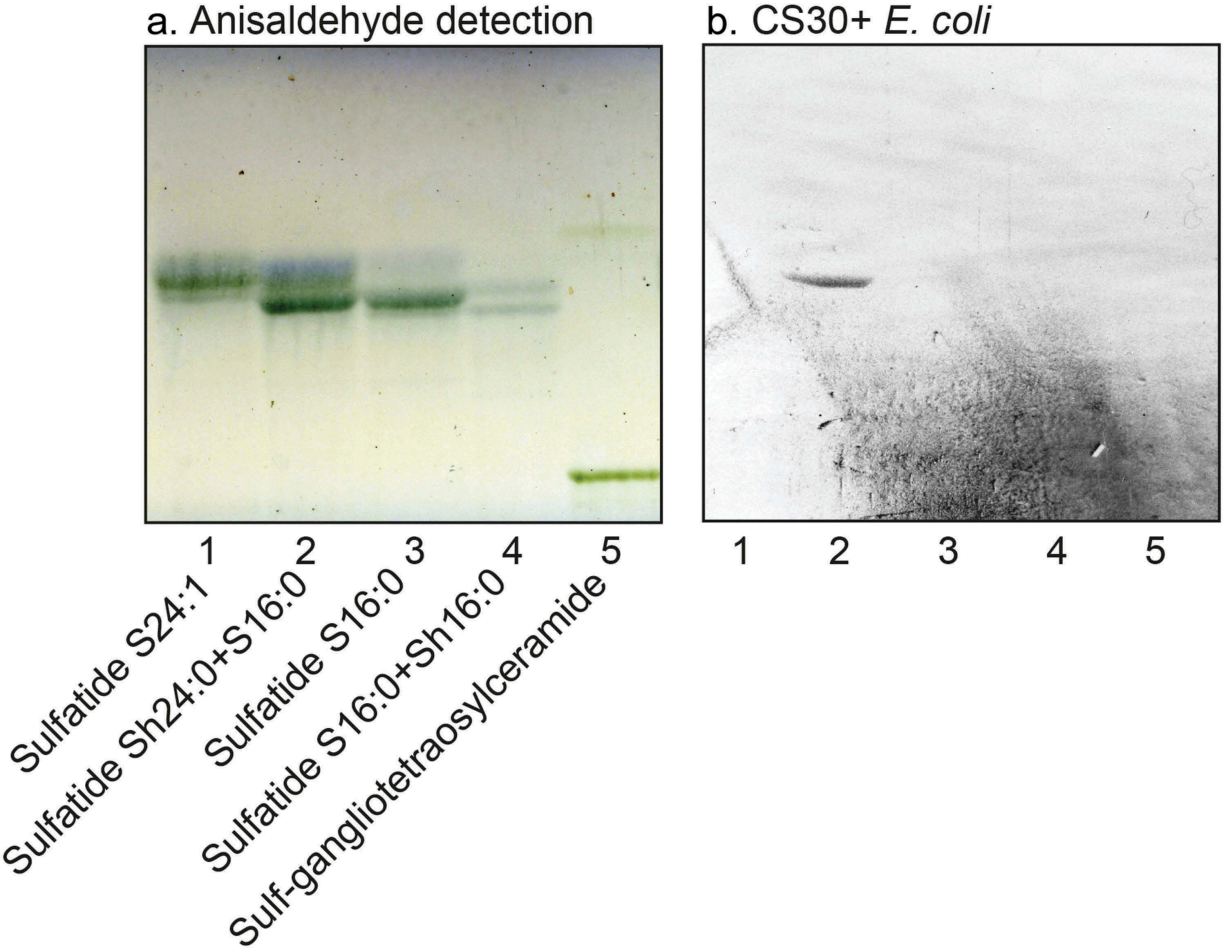
Binding of CS30 expressing *E. coli* to sulfatides with variant ceramides. Thin-layer chromatogram detected with anisaldehyde (a), and autoradiograms obtained by binding of the CS30 expressing *E. coli* strain E873 (b). The glycosphingolipids were separated on aluminum-backed silica gel plates, using chloroform/methanol/water 65:25:4 (by volume) as solvent system, and the binding assays were performed as described under “Materials and methods.” Autoradiography was for 12 h. The lanes were: Lane 1, sulfatide (SO_3_-Galβ1Cer) with d18:1–24:0 ceramide, 2 μg; Lane 2, sulfatide with d18:1-h24:0 ceramide and d18:1–16:0 ceramide, 2 μg; Lane 3, sulfatide with d18:1–16:0 ceramide, 2 μg; Lane 4, sulfatide with d18:1–16:0 ceramide and d18:1-h16:0 ceramide, 2 μg; Lane 5, sulf-gangliotetraosylceramide (SO_3_-3Galβ3GalNAcβ4Galβ4Glcβ1Cer), 2 μg.

**Figure 7.**
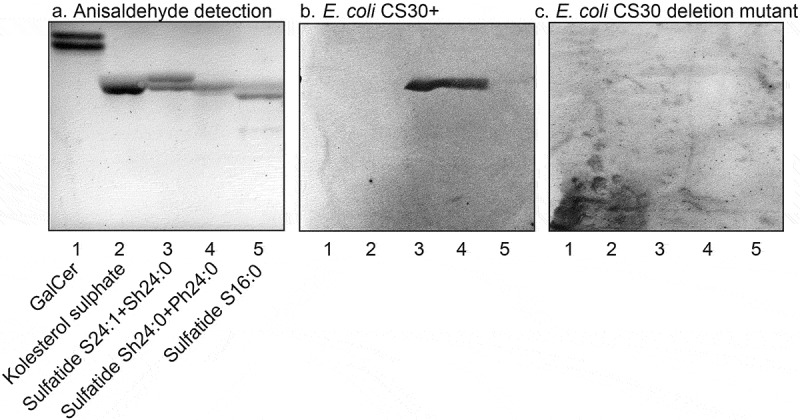
Binding of CS30 expressing *E. coli* to sulfatides with variant ceramides. Thin-layer chromatogram detected with anisaldehyde (a), and autoradiograms obtained by binding of the CS30 expressing *E. coli* strain E873 (b), and the mutant *E. coli* strain E873Δ*csmA*, lacking the major subunit CsmA (c). The glycosphingolipids were separated on aluminum-backed silica gel plates, using chloroform/methanol/water 60:35:8 (by volume) as solvent system, and the binding assays were done as described under “Materials and methods.” Autoradiography was for 12 h. The lanes were: Lane 1, galactosylceramide (Galβ1Cer), 4 μg; Lane 2, cholesterol sulfate, 4 μg; Lane 3, sulfatide (SO_3_-Galβ1Cer) with d18:1–24:1 and d18:1-h24:0 ceramide, 2 μg; Lane 4, sulfatide with d18:1-h24:0 and t18:0-h24:0 ceramide, 2 μg; Lane 5, sulfatide with d18:1–16:0 ceramide, 2 μg.

A number of sulfatides were also isolated from the total acid fractions of human stomach and human meconium by a series of Iatrobeads column chromatographies. Fractions obtained were pooled according to mobility on thin-layer chromatograms and binding of CS30+ ETEC, and characterized by LC-ESI/MS. Thereby a collection of sulfatides with variant ceramide composition was compiled. When binding of CS30 expressing ETEC to these sulfatides with different ceramides was examined there was a clear preferential binding to sulfatide with d18:1-h24:0 ceramide (Figure 6(b), lane 2; Figure 7(b), lanes 3 and 4). Again there was no binding by the mutant ETEC strain unable to express CS30 (Figure 7(c)).

A further observation from these binding studies was that the CS30 expressing ETEC did not recognize glycosphingolipids related to sulfatide, as galactosylceramide (Galβ1Cer; Figure 7, lane 1) and sulf-gangliotetraosylceramide (SO_3_-3Galβ3GalNAcβ4Galβ4Glcβ1Cer; Figure 6, lane 5). The absence of binding to galactosylceramide demonstrates that the sulfate moiety is necessary for the binding process. Furthermore, the non-binding of sulf-gangliotetraosylceramide shows that the ceramide is involved in the binding process, either as a part of the binding epitope, or by giving an optimal presentation of the SO_3_-3Galβ moiety.

### Inhibition studies

**Figure 8.**
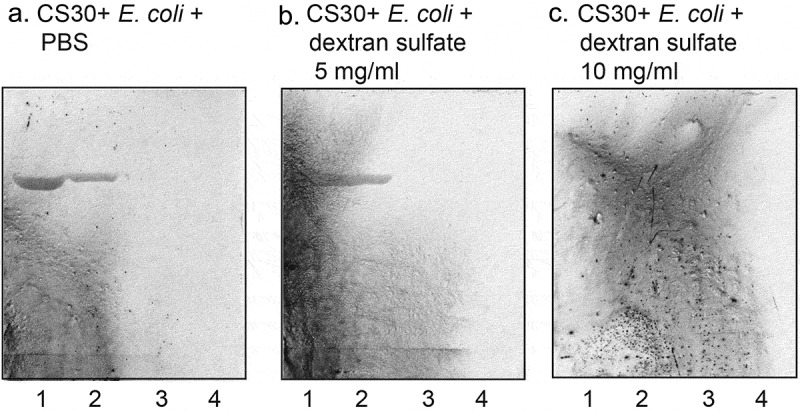
Effect of preincubation of CS30 expressing *E. coli* with saccharides. CS30 expressing *E. coli* were incubated with dextran sulfate (5 mg/ml and 10 mg/ml) in PBS for 2 h at room temperature. Thereafter the suspensions were utilized in the chromatogram binding assay. (a) binding of CS30 expressing *E. coli* alone, (b) binding of CS30 expressing *E. coli* incubated with heparan sulfate (5 mg/ml), and (c) binding of CS30 expressing *E. coli* incubated with heparan sulfate (10 mg/ml). The lanes were: Lane 1, sulfatide (SO_3_-Galβ1Cer), 4 μg: Lane 2, sulfatide, 2 μg: Lane 3, sulfatide, 0.8 μg: Lane 4, sulfatide, 0.4 μg. The glycosphingolipids were separated on aluminum-backed silica gel plates, using chloroform/methanol/water (60:35:8, by volume) as solvent system, and the binding assays were performed as described under “Materials and methods.” Autoradiography was for 12 h.

Finally, the ability of soluble saccharides and anionic polysaccharides to interfere with the binding of CS30 expressing ETEC to sulfatide was examined by incubating the bacteria with the saccharides before binding to a serial dilution of sulfatide on chromatograms. The interaction of CS30 expressing ETEC with sulfatide was abolished by incubation with dextran sulfate at 10 mg/ml (Figure 8(c)), whereas 5 mg/ml (Figure 8(b)) had no blocking effect. No inhibtion was obtained by incubating the bacteria with dextran, sodium octadecyl-sulfate, or galactose-4-sulfate (data not shown).

## Discussion

Adhesion of microbes to target cells is an important step in the infection process, allowing an efficient delivery of toxins and other virulence factors close to the cell surface. Potential host receptors, the majority of which are glycoconjugates, have been identified for a large number of bacteria [29]. In this study, we investigated the carbohydrate binding specificity of the novel CF CS30 by binding of CS30 expressing ETEC to a large number of variant glycosphingolipids. We thereby found a highly specific binding of CS30 to the glycosphingolipid SO_3_-3Galβ1Cer, also known as sulfatide. A preferential binding of CS30 to sulfatide with d18:1-h24:0 ceramide was obtained, and this ceramide species of sulfatide was isolated from human small intestine and characterized by mass spectrometry and antibody binding.

Sulfatide is also recognized by the nonfimbrial ETEC CF CS6 [11]. The CFA/I fimbriae, on the other hand, and also the highly homologous CS1 and CS4 fimbriae, bind to a battery of nonacid glycosphingolipids, as *e.g*. neolactotetraosylceramide, the H5 type 2, Le^a^ and Le^x^ pentaosylceramides [10]. Binding to these glycosphingolipids is mediated by CfaB, *i.e*. the major subunit of CFA/I [10], whereas the minor CFA/I subunit CfaE binds to gangliotetraosylceramide (asialo-GM1) [29].

Sulfatide recognition has also been reported for other pathogens, as *e.g. Mycoplasma pneumoniae* [30], *Bordetella pertussis* [31], and *Helicobacter pylori* [32,33]. In addition, sulfatide is recognized by the neutrophil-activating protein of *H. pylori* [34].

Interestingly, the crystal complexes of the *E. coli* FimH, FedF, and F17 G fimbrial adhesins with their respective carbohydrate ligands have in all three cases also highly charged regions in complex with sulfate in the vicinity of the reducing end carbohydrates of the ligands in their carbohydrate binding sites [35]. High mutation rates involving arginines and lysines was found in the two ETEC adhesins (10 in 17 of the F17 G, and 6 in 8 of the FedF lectin domains, respectively), and it was speculated that this may be a functional adaptation among ETEC strains allowing the bacteria to bind to carbohydrate receptors that are increasingly modified with negative charges downstream the intestinal tract.

Although ETEC is a highly heterogenous pathogen it has been shown that a mixture of prevalent antigens, such as CFs, can confer broad protection [36–39]. In addition, cross-reactive antibodies against related CFs are protective [40,41]. CS30 belongs to the Class1b CF group, also encompassing CS12, CS18, CS20, CS26, CS27, and CS28, four of which (CS26, CS27, CS28, and CS30) are relatively recently discovered. The porcine CF F6 (987P) is related to the Class 1b group, sharing more than half of the amino acid sequence of the major subunit of FasA (987P) with the major subunit of CS30 (CsmA) [9]. Interestingly, F6 fimbriae also bound to sulfatide [15,42], but this binding was occasional, in contrast to the high affinity binding to lacto/neolacto sequences obtained with the F6 fimbriae [15].

Whether a Class 1b CFs should be considered as a part of a future ETEC vaccine depends on the prevalence of Class 1b CFs, and the possibility of inducing cross-reactive antibodies, both of which have not yet been fully determined. The fact that CS30 is homologous to F6 and, as shown here, binds to sulfatide, the major acid glyosphingolipid of both human [27] and porcine [43] small intestine, makes it tempting to speculate that CS30 positive strains can infect multiple hosts.

## Abbreviations

CF: colonization factor
ETEC: enterotoxigenic *Escherichia coli*
LC-ESI/MS: liquid chromatography/electrospray ionization mass spectrometry
